# Detect and Trace: An Australian Field Trial Using Machine-Learning Tools to Combat Illegal Wildlife Trade

**DOI:** 10.3390/ani16050731

**Published:** 2026-02-26

**Authors:** Phoebe Meagher, Joseph Cincotta, Ha Tran Hong Phan, Kaikai Shen, Brad Dolman, Kate J. Brandis, Daniele Pelliccia, Christopher M. Poole, Kimberly Vinette Herrin, Justine K. O’Brien, Brendan E. Allman, Debashish Mazumder, Patricia S. Gadd, Vanessa Pirotta

**Affiliations:** 1 Taronga Institute of Science and Learning, Taronga Conservation Society Australia, Mosman, NSW 2088, Australia; jcincotta@zoo.nsw.gov.au (J.C.);; 2 Centre for Ecosystem Science, School of Biological, Earth and Environmental Sciences, University of New South Wales, Kensington, NSW 2052, Australia; kate.brandis@unsw.edu.au; 3 Rapiscan Systems Ltd., Homebush, Sydney, NSW 2041, Australia; 4 Department of Climate Change, Energy, The Environment and Water, Environmental Crime Section, Gadigal Country, Sydney, NSW 2000, Australia; 5 Instruments & Data Tools Pty Ltd., Vermont, VIC 3133, Australia; 6 Radiation Analytics Pty Ltd., Sunshine Coast, QLD 4551, Australia; 7 Australian Nuclear Science and Technology Organisation, Lucas Heights, NSW 2234, Australia

**Keywords:** wildlife, trade, technology, AI, artificial intelligence, wildlife trade

## Abstract

The illegal wildlife trade is a global problem that continues to harm individuals, wildlife populations, ecosystems, and humans at an increasing rate. While efforts are underway globally to address the issue through a coordinated approach, the testing of new technologies in real-world settings remains limited. Here, we present the outcomes of an opportunistic Australian trial that tested two machine-learning tools during real-world seizures, including associated radiation-exposure safety data. During the seven-month trial, 116 animals were intercepted, representing reptiles and crustacea across five Genera: *Tiliqua*, *Egernia, Oedura, Chelodina*, and *Euastacus*. Of the 18 seized consignments, totalling 48 parcels, scanned through the RTT^®^110 CT X-ray baggage scanner, automated AI detected smuggled wildlife 56% of the time using the most successful algorithm (AT.3), and captured 48 high-resolution 3D X-ray images, which allowed identification of concealed wildlife. In addition, 33 *Tiliqua* sp. were scanned using the Olympus Vanta pXRF and the data analysed using previously published machine-learning provenance models. Common blue-tongue lizards (*Tiliqua scincoides*) were less likely to be wild-caught than shingleback lizards (*Tiliqua rugosa*). Alongside expert statements, provenance results were provided to enforcement agencies. Following the trial, there was a significant reduction in the number of seized parcels being exported through postal pathways. This trial demonstrates the impact of integrating new technology to support intelligence-led enforcement processes and reduce wildlife trafficking.

## Simple Summary

While efforts are underway globally to combat illegal wildlife trade through a coordinated approach, testing of new technologies in real-world settings remains limited. Here, we present the outcomes of an opportunistic Australian trial that tested two machine-learning tools during real-world seizures, including associated radiation-exposure safety data. We demonstrate that machine learning technology can have a meaningful impact when used alongside law enforcement to reduce the illegal trade of wildlife.

## 1. Introduction

The illegal wildlife trade (IWT) represents one of the most pressing threats to global biodiversity, contributing to population declines, ecosystem disruption, poor animal welfare and the spread of zoonotic diseases [1,2,3,4]. Australia is home to many unique species prized overseas in the exotic pet trade [5,6,7,8]. Australian wildlife trade is primarily regulated by the federal Environment Protection and Biodiversity Conservation Act 1999 (EPBC Act), which strictly controls the import/export of native and international species and enforces international CITES (Convention on International Trade in Endangered Species) obligations. State and territory laws impose additional controls on local wildlife, while the Biosecurity Act 2015 regulates entry risks [9]. Despite strict national legislation, Australian species continue to be found outside of the country in large numbers, with a recent analysis identifying 170 endemic herpetofauna (reptile and amphibian) species in international trade [6]. This is due to high international demand and prices, involvement of organised criminal syndicates, gaps in detection, low penalties imposed by the legal system and the ease of concealment, transport and wild collection of live specimens [5,10,11]. The domestic reptile pet trade legislation is inconsistent and minimally regulated, with the current process for ensuring the captive-bred origin of pet species relying on an honour-based reporting system that opens the possibility of wild-caught individuals being traded and sold online as captive-bred [8].

Traditional enforcement and IWT monitoring methods are becoming more coordinated; however, technological innovations have often been overlooked due to a lack of pathways for real-world testing and application [12]. Illegal wildlife trade can be disrupted at various stages of the transaction pathway, from acquisition (facilitated by wild poaching or illegal domestic breeding), through to detection, as wildlife are smuggled into and out of countries. The integration of technology at multiple points along the transaction pathway can be used alongside each other to support and complement outcomes. While wildlife forensic technology is a growing research area, implementation into enforcement practices is low due to resources, training and collaboration pathways [13,14].

Detection tools have increased considerably in the past decade, from the use of biosecurity dogs [15] and human-analysed 2D X-ray scans of mail and luggage [16,17], to volatilome profiles and the development of e-noses for remote wildlife detection [18,19]. Artificial intelligence (AI), drones, satellite remote sensing, Internet of Things (IoT) devices, blockchain, phone-based apps, Spatial Monitoring and Reporting Tools (SMART) and autonomous systems are among the emerging tools revolutionising illegal wildlife detection and surveillance [14,17,20,21,22]. Cyber-crime detection through automated analysis of internet traffic and social media is also becoming a common tool as IWT moves to online platforms [18,19,20,21,22]. Emerging 3D X-ray systems, like the Real Time Tomography 110 (RTT^®^110) system (Rapiscan Systems), are reported to offer enhanced detection capabilities, reduced false positives and improved screening efficiency [23]. Despite these advances, gaps remain in detection and provenance tracing capabilities [24], demonstrated by the high number of Australian species in the illegal wildlife trade [6].

Tracing the origins of trafficked wildlife has become a conservation priority [25,26]. Determining whether an animal has been collected from the wild is important for assessing conservation risk at the regional or species level, as well as for increasing potential penalties imposed by authorities [27,28,29]. Many species are traded under the guise of captive breeding when they have been illegally poached from the wild or when their progeny is derived from wild-caught animals [30,31,32]. Morphometrics have been used for the identification of provenance [25,30] but have limitations. Phenotypic characteristics can vary with age, sex, environmental pressures and selective breeding [33,34,35,36] or be common to a genus that can have large distribution ranges, making provenance hard to pinpoint. In some cases, smuggled wildlife is not intact or the whole animal is not available, making morphometrics unreliable [37]. Finding and evaluating robust, quick and effective methods of determining the origin of seized wildlife is crucial for combating IWT, and has resulted in varying techniques for determining origin, including stable isotope analysis, elemental analysis and molecular genetic techniques [26,38,39,40]. Molecular genetic techniques are mostly reliant on DNA extraction from non-degraded tissue and on the availability of samples from a known origin, which can be impractical [40]. Comparison studies comparing stable isotope analysis with elemental analysis have shown greater accuracy with elemental analysis [41,42]. Elemental analysis through XRF has proven successful for determining provenance in biological tissue using ITRAX scanners [41,42]; however, the ITRAX scanner can only analyse parts of biological tissue and is lab-based [41,42,43]. More recently, portable X-ray fluorescence (pXRF) has been tested as a provenance tracing tool on biological tissue [42,44,45]. The advantages of pXRF are that it is portable, quick and non-invasive; animals can be scanned alive in less than a minute and released with no samples taken or negative impacts [44,45]. Published models and methods for provenance testing using the pXRF are available for shinglebacks (*Tiliqua rugosa*) and Common blue-tongue lizards (*Tiliqua scincoides)* [40].

The aim of our research was to test the practicalities and limitations of two new machine learning tools as part of an end-to-end wildlife confiscation pathway with real-world seizures alongside the Commonwealth Environmental Crime Team in the Department of Climate Change, Energy, the Environment and Water (DCCEEW). The two tools selected were explicitly chosen for their novelty and complementarity in an end-to-end operating procedure. The RTT^®^110 3D X-ray Tomography Machine (Rapiscan Systems) was used for testing detection image algorithms [46] on concealed wildlife, and the Vanta M-Series (Evident corporation) portable X-ray fluorescence (pXRF) device was used to trial *Tiliqua* sp. elemental provenance models on seized lizards [40]. The pXRF provenance results are published separately in Brandis et al. 2025 [40], alongside details of the model development. This paper provides an overview of the end-to-end process, outcomes, radiation safety data and recommendations.

## 2. Materials and Methods

Between July 2023 and January 2024, we trialled an end-to-end processing pathway for seized wildlife as part of assisting federal officers from the Environmental Crime Unit of the Department of Climate Change, Energy and Environment and Water (DCCEEW) as part of Operation Blade. We had the opportunity to test two recently published machine-learning technologies on parcels and specimens seized by Australian Federal Officers suspected of concealing wildlife destined for export out of Australia via postal pathways. The first machine learning technology used the RTT^®^110 3D X-ray Tomography Machine (RTT^®^110) (Rapiscan Systems, Torrance, CA, USA) and methods developed by Pirotta et al. [46] to identify concealed wildlife. The second machine learning technology used the Evident Vanta M Series portable X-ray fluorescence (pXRF) analyser (Evident Corporation, Waltham, MA, USA), and methods developed by Brandis et al. [40] to identify the provenance of seized *Tiliqua* sp. lizards. The materials and methods described here are an overview of the trial. Details of model development can be found at Pirotta et al. 2022 [46] and Brandis et al. 2025 [40].

### 2.1. End-to-End System Overview

**Figure 1:**
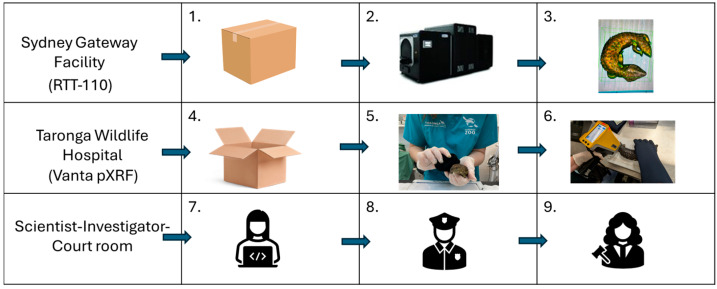
Step-by-step overview schematic of the end-to-end technology-assisted processing of seized wildlife during the trial, July 2023–January 2024. 1 = unopened parcel. 2 = RTT^®^110 scan. 3 = 3D-generated image. 4 = opened parcel. 5 = veterinary examination. 6 = pXRF scan. 7 = Data analysis. 8 = Special Investigator evidence collection. 9 = Court hearing.

As part of the existing outbound (international) mail screening of packages at the Sydney Gateway Facility (SGF), suspicious parcels identified by human inspection were flagged by Australian Border Force personnel to DCCEEW Wildlife Crime Special Investigators. Flagged packages remained unopened and scanned at the SGF using the RTT^®^110 3D X-ray Tomography Machine (RTT^®^110) to generate 3D images of each item. Packages were scanned opportunistically as identified. Packages were placed in an upright position and scanned once. The image generated from the item was then visualised by manipulating the image on the screen to inspect the internal contents and identify the type of wildlife within it (steps 1–3, Figure 1). These 3D images were then sent to the Taronga Wildlife Hospital to assist in the preparation of appropriate animal handlers, housing and veterinary treatment. Within 48 hours of wildlife being confirmed within a parcel, investigators would transport parcels to Taronga Wildlife Hospital. Unboxing would occur under video surveillance in the presence of federal investigators, Taronga reptile handlers, veterinary officers and research scientists at Taronga Wildlife Hospital. Once appropriate evidence was collected, trained animal handlers and veterinary staff would remove wildlife from the packaging they were restrained or concealed in. During the trial, examples of concealment included wildlife hidden in socks, stockings, plush and plastic toys, backpacks, printers, and potato chip, cereal and chocolate containers.

Species identification of seized wildlife was confirmed onsite by expert herpetologists, examined by veterinary staff, given identification numbers, and weighed. Each shingleback lizard *(T. rugosa*) and common blue-tongue lizard *(T. scincoides*) was placed individually on a custom-built scanning platform with the tail held over an embedded silica disc [40]. Lizards were scanned at the base of the tail, where it meets the body, just below the hind leg hip joints (Taronga Animal Ethics approval 3d/12/20) (steps 4–6, Figure 1). Thirty-three lizards were scanned with the pXRF analyser and had provenance models applied as per Brandis et al. 2025 [40]. Provenance results, alongside images produced by the RTT^®^110 and expert statements from herpetologists and veterinary staff, were compiled into reports and sent to wildlife crime special investigators. Information from these reports was used as evidential support in the case presented to the courts (steps 7–9, Figure 1).

### 2.2. Rapiscan RTT^®^110 Technical Methods

The RTT^®^110 (Rapiscan Systems) uses a stationary gantry with multiple X-ray sources to produce high-resolution 3D X-ray CT images in real-time [47], which can be manipulated to provide a 360-degree view of an animal. This system is used at Australian international borders and at airports and mail-cargo facilities worldwide for explosive-detection screening. This paper presents the first real-world trial of the RTT^®^110 with live wildlife cases. The RTT^®^110 system’s standard reconstruction software generates volumetric and morphological images and computes the intensity distribution, size and shape. These features are then used in the machine learning process to develop the detection model. The models used classify the labelled components into wildlife subcategories (‘Lizard’, ‘Fish’ and ‘Birds’) with a detection rate of 82% and a false positive rate of at 1.6% [46]. The Animal Trafficking detection algorithm AT.2 [46] was based on a Random Forest algorithm implementation [48], which aggregated the classification results from decision trees built from random samples of the training data to produce the detection results. For algorithm AT.3, volumetric images of radiodensity for scanned objects were reconstructed using the RTT^®^110 system’s standard reconstruction software. Thresholding on the intensity values to extract the volumes within the range of organic materials, followed by a morphological opening operation were performed. The extracted volumes were then labelled by a connected component analysis to form 3D segmented blobs. Maximum intensity projection (MIP) [49] was applied along the three orthogonal axes of the segmentation blobs, rendering 2D projections of size 128 × 128 and then a pre-trained MobileNetV2 convolutional neural network (CNN) [50] was used to extract a feature vector of size 160 which are then concatenated and passed into a stack of five fully connected layers and a softmax output layer [51] for the wildlife subcategory classification. AT.3 enhanced the ability to detect and generalise from sample data over AT.2. The implementations for both algorithms were as described by Pirotta et al. [46] using the original datasets for training and validation of the respective models.

### 2.3. Rapiscan RTT^®^110 Radiation Exposure

The Rapiscan RTT^®^110 is a hold baggage scanner with a tunnel diameter of ~100 cm. It operates at 160 kV accelerating voltage (a conventional bone X-ray is at 60 kV). The security scanner’s higher voltage is necessary to generate X-ray energies capable of penetrating the contents of bags, which may include highly absorbing metals. At the centre of the radiation-shielded tunnel, the RTT^®^110 has a collimated curtain X-ray beam through which the bags pass. This beam is ~2 mm wide and scanning at 0.5 m/s means that any 2 mm feature is only exposed for 4 ms. Experimental measurement of energy levels was not taken on the RT110; rather, energy levels were derived using the device’s certified operating characteristics.

### 2.4. Portable X-Ray (pXRF) Fluorescence Technical Methods

The Vanta pXRF analyser (Evident corporation) contains a 4-watt X-ray tube with tungsten (W) anode, 8–50 keV, silicon drift detector with three beam energies (10, 40, and 50 keV). The Vanta pXRF analyser provides elemental concentrations as percentages for 42 elements, calculated by the manufacturers’ on-board algorithm. Only shingleback (*T. rugosa*) and common blue-tongue lizards (*T. scincoides*) were scanned with the Vanta pXRF based on the availability of previously published models. Lizards were scanned using the methodology as published by Brandis et al. 2025 [40] and had previously published predictive models applied to data. Models reported to have accuracies of 81% for distinguishing captive-bred from wild individuals for common blue-tongue lizards (*T. scincoides*) and 83% for shingleback lizards (*T. rugosa)* [40]. For further details on method set-up, model development and underlying classifiers, see Brandis et al. 2025 [40].

### 2.5. pXRF Radiation Exposure

To ensure the specimens are not exposed to dangerous levels of cumulative radiation during the end-to-end trial and to provide radiation exposure estimates to industry, scintillation gamma/beta radiation detectors were used to measure radiation after irradiation with the Vanta pXRF analyser.

Four common blue-tongue lizards (*T. scincoides*) specimens were provided for the radiation exposure study through Taronga Wildlife Hospital. The specimens had died from unrelated causes and had been frozen and thawed prior to exposure to radiation. Only *T. scincoides* specimens were used in the radiation testing due to availability of specimens and being a representative group exposed to both RTT^®^110 3D X-rays and Vanta pXRF radiation during the end-to-end trial. We would recommend similar dosimetry experiments to be performed on other groups of wildlife if they are to be exposed to both forms of radiation.

The carcasses were exposed to irradiation using the Vanta pXRF with Prolene 6 μm measurement window and set to a 60 s “3-beam GeoChem (50 kV)” scan protocol setting as per Brandis et al. [40] comprising three beam scans (beam 1. V = 10 kV I = 78.03 μA, no filter, t = 20 s; beam 2. V = 40 kV, I = 60.75 μA, Al 2 mm filter, t = 20 s; and beam 3. V = 50 kV, I = 60.8 μA, Cu 0.350 mm filter, t = 20 s) as would be used on real seized specimens. 

Before irradiating the carcasses, a water-equivalent phantom of Solid Water HE (Gammex Technology Corporation, Wisconsin, United States) was irradiated using the same protocol and pXRF device. Scintillation detectors were used to measure radiation levels on the phantoms: before exposure to the XRF beams to measure ambient radiation; during exposure to the XRF beams to measure direct surface exposure; and again after exposure to measure any ambient radiation. All measurements were used as baselines for comparison with soft tissue.

Scintillation detectors were placed directly on the skin for 30 s immediately after XRF exposure. Following the surface measurements, each specimen was transversely sliced at the centre of the point where the surface measurement was taken. At a depth of 10 mm from the sample surface, cross-sectional dosimetry measurements were taken using the same approach, providing insights into ambient radiation at different depths.

To determine energy deposition within soft tissue, we modelled the X-ray tube spectra using SpekPy [52], first calculating the dose (air kerma) at the sample surface and normalising it to the measured dose during the phantom XRF beam exposure. Then we calculated the dose within the keratin layer, applied normalisation, and finally calculated the dose after the keratin layer into a 20 mm-thick water layer (simulating the thickness of the soft tissue). Modelling assumed keratin average composition C_27_H_57_N_8_O_17_S_1_, density 1.32 g/cm^3^, and a keratin layer thickness of 0.5 mm.

Species-specific organ weighting factors ($w_{T}$) for reptiles remain unavailable in the literature [53], precluding a conventional calculation of effective dose (E) based on organ-specific absorbed doses; however, we adopted a pragmatic approach: we assumed that the measured localised air kerma (in mGy) is directly equivalent to the effective dose in mSv (i.e., $E\approx D_{\mathrm{local}}$ with a conversion factor of 1.0 mSv per mGy). This assumption is consistent with approaches used in non-human biota dosimetry when organ-specific data are lacking [54,55] and is justified here by the small spatial extent of irradiation relative to body size.

To model the exposure geometry, we defined the irradiated volume based on the specimen’s physical dimensions and the effective X-ray beam footprint. Specimens were approximated as rectangular prisms with mean dimensions of 100 mm (length) × 20 mm (width) × 20 mm (depth), representative of the body profile of small to medium-sized lizards. The pXRF beam, characterised by a conical emission profile, was modelled as having an incident cross-section of 15 mm × 15 mm and a depth equal to the specimen’s thickness (20 mm), ensuring full volumetric coverage of the irradiated region. The resulting irradiated volume ($V_{\mathrm{irr}}=15\times 15\times 20\;{\mathrm{mm}}^{3})$ corresponded to approximately 4.5% of the total specimen volume ($V_{\mathrm{specimen}}=100\times 20\times 20\;{\mathrm{mm}}^{3}$), confirming that the dose is highly localised. Under these assumptions, all subsequent dose calculations were constrained to this irradiated sub-volume, and the measured localised air kerma, normalised to the surface measurement detected using the scintillator and interpreted as representative of the absorbed dose within this region. No further spatial or organ-specific weighting was applied.

## 3. Results

### 3.1. Detection: RTT^®^110 3D X-Ray Tomography

**Figure 2:**
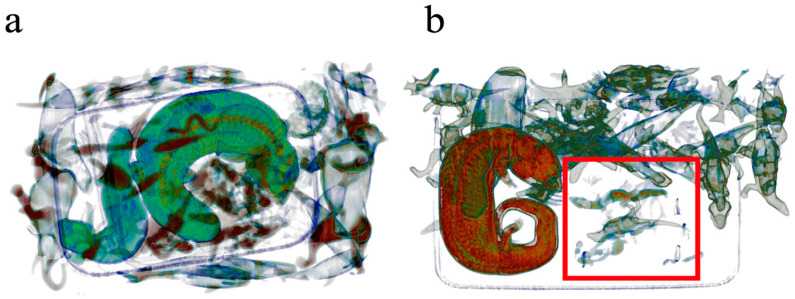
Images produced using the Rapiscan RTT^®^110 3D X-ray imaging machine. (**a**) Detection of two *Tiliqua rugosa* in a bag full of items, including toys. (**b**) *Tiliqua scincoides*. and several *Oedura castelnaui* (red square).

During the trial, 48 separate parcels were scanned once through the RTT^®^110, producing 48 3D high-resolution images. The images produced clearly show the *Tiliqua* specimens against plastic toys (Figure 2a,b). It was incidentally observed, in one consignment, that five smaller northern velvet geckos (*Oedura castelnaui*) were not as visually obvious on images produced (red box—Figure 2b) compared to the *Tiliqua* specimen. Comparative detection capability of the algorithm between lizard species was not quantified as part of this study but is worthy of note. Detection of ‘lizards’ varied from 21% for algorithm AT 2 and up to 56% for algorithm AT 3 (Table 1). To assess the statistical significance of the improvement in accuracy from AT.2 to AT.3, a one-tailed Z-test was performed against the null hypothesis that the accuracy of AT.3 is not higher than that of AT.2. We found the difference in the accuracies between AT.3 and AT.2 is statistically significant with *p* = 0.00018.

### 3.2. RTT^®^110 Radiation Exposure

The manufacturer-calculated radiation exposure for the RTT^®^110 is conservatively less than 5 mSv per scan. This was calculated using current, energy of X-ray (keV), exposure time and distance from the X-ray source (dose decreasing with the inverse square of distance).

### 3.3. Wildlife Seizure 

**Figure 3:**
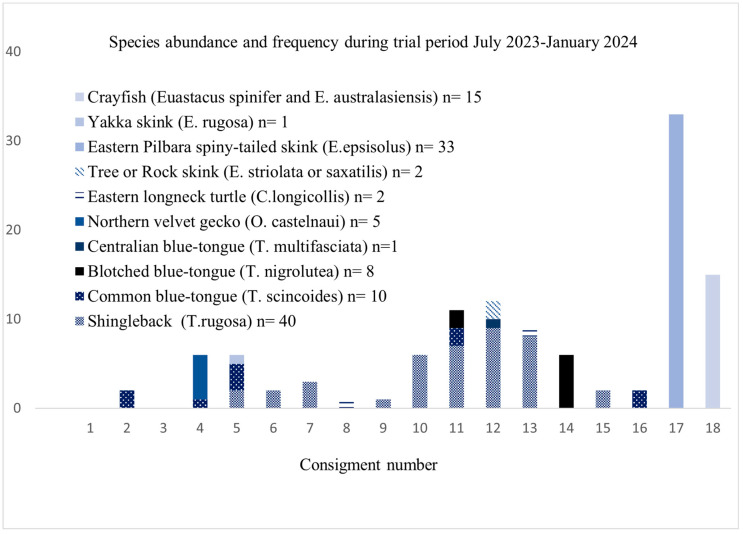
Frequency and number of individuals by species seized during the trial period by consignment (consignments varied in number of parcels).

During the trial period, 116 animals were seized from 18 consignments consisting of 48 individual parcels being exported out of the country through postal pathways. Eighty-seven per cent were reptiles covering at least nine different species. The most frequently seen species across the trial period included shingleback lizards (*Tiliqua rugosa*) in 9 of the 18 consignments and common blue-tongue lizards (*Tiliqua scinoides*) found across 5 of the 18 consignments. Shingleback lizards (*T. rugosa*) were the most abundantly found species during the trial period *(n* = 40), followed by Eastern Pilbara spiny-tailed skink (*Egernia epsisolus*) (*n* = 33), crayfish (*Euastacus spinifer* and *Euastacus australasiensis*) (*n* = 15) and common blue-tongue lizards (*T. scinoides)* (*n* = 10) (Figure 3). However, the spiny-tailed skinks and crayfish were only found in a single consignment each. Several species were only found in a single consignment including; centralian blue-tongue lizards (*Tiliqua multifasciatea*) *(n* = 1); northern velvet geckos (*Oedura castelnaui*) (*n* = 5); eastern longneck turtle (*Chelodina longicollis*) (*n* = 1); and tree or rock skinks (*Egernia striolata or saxatilis*) *(n* = 8). Blotched blue-tongue lizards (*Tiliqua nigroluta*) where found in two separate consignments (*n* = 8) (Figure 3). Eighty-three percent of all reptiles that came through Taronga Wildlife Hospital were successfully re-homed within the zoo system, 17% had to be euthanized due to health or welfare concerns, and only one lizard was found dead upon unboxing. All crayfish were dead on arrival.

### 3.4. pXRF Provenance Tracing

The data from the provenance tracing on 23 shingleback lizards (*T. rugosa*) and 10 common blue-tongue lizards (*T. scinoides*), scanned as part of the trial, have been included in the previous publication Brandis et al. 2025 [40]. A high-level summary table is presented here as an overview of trial results only (Table 2). Undetermined classification was assigned when the probability was less than 65% for assigning origin.

### 3.5. pXRF Radiation Exposure

The Vanta pXRF average ambient radiation levels during measurement were 0.2 mSv at the keratin surface and 10 mm below that post-exposure. However, direct surface exposure was measured at 227 mSv over the 60 s measurement period.

**Figure 4:**
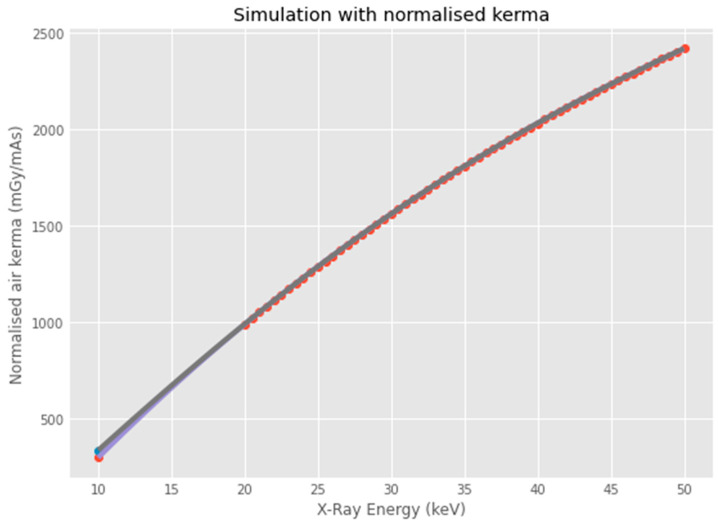
Graph of SpekPy [52] simulation of air-kerma produced by XRF irradiation. The SpekPy calculation threshold is 20 keV (red points); we extrapolate values from 10 keV to 20 keV to cover the range of energies used by the Vanta M pXRF (blue line).

Our Monte-Carlo-based attenuation model, normalised to a measured surface air kerma of 227 Gy (see Table 3 and Figure 4), demonstrated that the keratinous scales of lizards significantly attenuated low-energy pXRF. The modelled transmission through a 0.5 mm keratin layer (density: 1.32 g/cm^3^; composition: C_27_H_57_N_8_O_17_S_1_) reduces the incident photon flux by approximately 68.8%, resulting in a soft-tissue absorbed dose of 71 mGy, equivalent to 31.2% of the surface dose.

This value represents the localised absorbed dose delivered to the underlying soft tissue within the irradiated volume. To estimate the effective dose (E), we accounted for the spatial confinement of the pXRF beam relative to the total body mass. Assuming uniform dose distribution within this irradiated region and no significant scatter from surrounding tissues, the effective dose was calculated as:

(1) $$E=D_{\text{soft-tissue}}\times f_{\text{irradiated}\;\text{volume}}=71\;\mathrm{mGy}\times 0.045=3.2\;\mathrm{mSv}$$

## 4. Discussion

This trial highlights how integrating machine-learning tools into law enforcement pathways can have a real impact in reducing illegal wildlife trade. It further provides a snapshot of trade data and trends over the trial period, and a review of radiation exposure considerations when using these X-ray-based technologies.

### 4.1. Wildlife Seizures

Shingleback lizards (*T. rugosa*) were the most common and most abundant species found concealed for illegal export through the post during this trial. This supports previous analysis of Australian live seizures, where over half of smuggling attempts analysed involved shingleback lizards, but contrasts with species commonly seen for sale online [6], suggesting different species are at risk across different pathways. Other reptile species being trafficked during the trial included skinks (Scincidae), geckos (Diplodactylidae), and freshwater turtles (Chelidae), which are also commonly reported as coveted native species in the illegal wildlife export trade [6]. Additionally, one shipment of crayfish (Euastacus) was detected within the trial period. Crayfish are most likely smuggled for the pet trade [56], and while less commonly smuggled than reptiles, they represent the increasing occurrence and growing concern around invertebrates being smuggled out of Australia [57,58].

High survival rates at unboxing support the robustness of reptiles under these conditions, contributing to their appeal for international trafficking [59]. One lizard was dead on arrival, and 16% of lizards required euthanasia due to either the presence of respiratory symptoms or welfare concerns, including severe malnutrition, dehydration, or trauma, suggesting poor welfare conditions during holding and trafficking. This supports case studies that demonstrate that the average welfare experience of a wild animal being traded is negative and that most animals routinely experience negative states such as extreme hunger and thirst, pain, fear, and chronic stress [60].

### 4.2. Radiation Exposure

The reported radiation measurements from the manufacturer for the RTT^®^110 of 5 mSv are less than two years of natural background exposure and half the 10 mSv of a medical CT exposure. The speed and source distance of the RTT^®^110 favourably reduce the exposure [61]. To our knowledge, this is also the first study to quantify X-ray attenuation by reptilian keratin using physics-based dosimetry, revealing that ~69% of pXRF radiation is absorbed at the scale surface. This finding has direct implications for forensic traceability. By enabling accurate correction of surface-to-tissue dose ratios, our method demonstrates the safety of pXRF-based elemental fingerprinting in reptile wildlife forensics, allowing more precise determination of geographic provenance and detection of illegally traded reptiles without harming live specimens. The computed effective dose from a single pXRF measurement is equivalent to that from a clinical imaging procedure, such as an abdominal CT scan. Combined with the initial X-ray tomography exposure (at the time of initial detection), a whole-body exposure of approximately 8.2 mSv remains within the world-average range of annual natural background ionising radiation exposure [62]. While frequent and repeated exposure to this scanning protocol is not recommended, a single exposure does not represent a dose that would have any long-term impact on the animal. The surface air kerma of 227 mGy highlights the importance of radiation safety training, personal protective equipment, and safe operating procedures when handling and operating the Vanta pXRF.

### 4.3. RTT^®^110: Detection

This trial demonstrated the adaptability and complementary nature of 3D X-ray tomography for automatically detecting concealed wildlife during real-world seizures. It also highlights the importance of providing high-resolution images, which can be manipulated to inform personnel about items within packages before unboxing at wildlife care facilities. Information on potentially dangerous species, e.g., venomous snakes, enables staff to adequately and safely prepare to handle and treat them. An incidental finding of the trial revealed that smaller Diplodactylidae species (*O. castelnaui*) are more likely to pass undetected than larger *Tiliqua* sp. (*T. rugosa and scincoides*), although this was not quantified as part of this study. Experimentally testing differences in detection accuracy across lizard species would be an important area for future investigation.

The significantly improved detection accuracy of AT.3 compared to AT.2 can be explained by an additional 78 lizard data points added to the training set. Training data for detection models had reduced clutter compared to the presentation of real-world cases, which may have limited the detection effectiveness during the trial. Only scanned parcels already suspected of having concealed wildlife were scanned through the RTT^®^110, resulting in no negative controls during the trial, which may also be another limitation to the interpretation of results. We would recommend quantifying the impacts of different concealment methods on the accuracy of detection in future work. Data acquired from this operation can now be incorporated to train future models and, therefore, better reflect in situ samples. This also highlights the need to continue to update training data and models, given that traffickers may change how they conceal wildlife over time.

Whilst we did not directly compare the detection rate with other X-ray technologies, 3D systems are reported to offer enhanced detection capabilities, reduced false alarms and improved screening efficiency [23]. As this was an opportunistic trial of relatively low sample size, with both time pressures due to animal welfare and resources, making controls unavailable, conclusions and comparisons are limited. We would recommend a robust experimental blind test set of both negative controls and parcels containing wildlife tested across both 2D and 3D X-ray systems. The broader-scale rollout of these devices and algorithms requires additional resources and training to further explore solutions to address the welfare and repatriation of an increased number of animals resulting from higher detection rates.

### 4.4. pXRF: Tracing Provenance

At the conclusion of the trial period, provenance results from the Vanta pXRF analyser and associated *Tiliqua* sp. provenance algorithm were provided as intelligence to enforcement agencies. The data provided indicated that common blue-tongue lizards (*T. scincoides*) were less likely to be wild-caught compared to shingleback lizards (*T. rugosa*), and shingleback lizards are less likely to be captive-bred than common blue-tongue lizards. Nearly half the provenance results did return an ‘undetermined’ classification, which is determined by probabilities being less than 65% or the probability mean not exceeding the mean ± SD of the alternative classification of the 100 models run [40]. Several factors could result in low provenance probability, including time in a captive environment and keratin turnover rate, type of captive enclosure, e.g., indoor vs. outdoor, and diet of the animal [40]. We present the impact, limitations and considerations of the trial here, not details of model limitations and improvements, which can be found in previously published work by Brandis et al. 2025.

Testing the Vanta pXRF analyser alongside law enforcement in real-world seizure operations for the first time allowed a unique opportunity to explore the reality of using technology in high-pressure situations, when research aims and data collection often come second to evidence collection, human safety and animal welfare. Availability of trained staff to scan at short notice was a limiting factor to the number of lizards scanned as part of the trial. Staff trained to use the pXRF analyser were outside of the law enforcement team. Based on this trial, we would recommend that special investigators be trained in pXRF analyser use and safety, and have access to additional machines so as not to rely on external staff availability. This is true for running the provenance models, which, during the time of the trial, required the Vanta pXRF analyser to be transported off-site and given to model developers to run data. The authors of this paper are developing a cloud-based, real-time mobile phone application that can run the data through the models within minutes of scanning the animal to reduce this turnaround time.

The provenance results support anecdotal information that common blue-tongue lizards are more likely to be captive-bred, given the demand for captive-bred colour-morphs and well-established captive breeding husbandry [56,63,64]. Compared to blue-tongue lizards, shingleback lizards are currently more challenging to breed in captive settings [59,65], and easy to collect in the wild [7,66], with wild-caught colourations, such as those found in the Goldfields region of Western Australia, in high demand [56,59,66]. Providing robust, data-driven intelligence on provenance can provide authorities with information on pathways for acquisition for different species, and increase penalties handed down if animals are wild-caught [67,68]. While it may not necessarily meet the standard of proof by legal definitions, it does for scientific purposes [56,69] and can be included as intelligence in broader impact statements presented to the courts [56].

Evidence of animal cruelty, provided through veterinary reports, can also increase the severity of penalties handed down [68]. On completion of the trial, Taronga’s veterinary and expert statements were provided alongside provenance reports to federal investigators and used as evidential support to the brief of evidence during Operation Blade [9], which resulted in aggregated sentences of three years and six months in gaol, with a two-year non-parole period.

Understanding animal origin opens possibilities for repatriation pathways [41,70,71]; however, we are constrained by the unknown disease risks of releasing wildlife, particularly reptiles, back into the wild [72]. We recommend a comprehensive disease risk assessment for the repatriation of commonly trafficked Australian species, and the feasibility of long-term holding and soft-release options for high-risk species.

## 5. Conclusions

This opportunistic end-to-end trial demonstrates the potential application and impact of technology and machine learning models in disrupting IWT. The trial also demonstrates the power of collaboration across science, industry and law enforcement. While combating wildlife trade is complex, testing new tools alongside each other in on-ground scenarios is crucial for progress. Both the RTT^®^110 and Vanta pXRF analyser have demonstrated their impact on fighting IWT in Australia in real-world scenarios and make valuable additions to the ever-increasing toolbelt of science-based tools in this fight to reduce the illegal trade of native species in Australia.

## Figures and Tables

**Table 1:** Rapiscan RTT^®^110 scan results during the July–December 2023 trial testing algorithm AT 2 and AT 3. (successful detections/number of parcels scanned).

Scan Date (DD/MM/YYYY)	Consignment Number	Number of Parcels	No. Images	Algorithm 1: AT 2	Algorithm 2: AT 3
21 December 2023	18	1	1	Failed	Failed
7 December 2023	17	4	4	Failed	Partial (2/4)
5 December 2023	16	1	1	Successful (1/1)	Successful (1/1)
19 October 2023	15	3	3	Failed	Partial (1/3)
11 October 2023	14	1	1	Successful (1/1)	Successful (1/1)
29 September 2023	13	9	9	Partial (2/9)	Partial (2/9)
26 September 2023	12	3	3	Failed	Partial (1/3)
14 September 2023	11	5	5	Partial (1/5)	Successful (5/5)
8 September 2023	10	5	5	Partial (1/5)	Partial (2/5)
5 September 2023	9	1	1	Successful (1/1)	Successful (1/1)
1 September 2023	8	3	3	Failed	Failed
31 August 2023	7	2	2	Failed	Successful (2/2)
25 August 2023	6	2	2	Failed	Partial (1/2)
21 August 2023	5	3	3	Partial (1/3)	Successful (3/3)
18 August 2023	4	1	1	Failed	Successful (1/1)
11 August 2023	3	1	1	Successful (1/1)	Successful (1/1)
7 August 2023	2	1	1	Failed	Successful (1/1)
21 July 2023	1	2	2	Partial (1/2)	Successful (2/2)
Total		48	48	20.83% success	56.25% success

**Table 2:** Summary of provenance results from trial (originally published data with 95% CI’s reported in Brandis et al. 2025 [40]).

Predicted Origin	Species	
	Blue-tongue lizards, *T. scincoides* (*n* = 10)	Shingleback lizards, *T. rugosa* (*n* = 23)
Wild	10%	26%
Captive	50%	34%
Undetermined	40%	39%

**Table 3:** Scintillator measurements were taken for each treatment.

Treatment	Surface μSv	10 mm Depth μSv
Control 1 direct XRF exposure	227,000	NA
Control 2 no XRF exposure	177.2	156.09
Specimen 1	189.4	168.7
Specimen 2	175.1	203.9
Specimen 3	174	272.4
Specimen 4	250.5	-

## Data Availability

The data presented in this study may have restrictions due to the sensitive and legal nature of the data, due to the data being part of an ongoing study, and due to resource limitations. All requests will be considered and subject to approval by the Department of Climate Change Energy Environment and Water and/or Rapiscan. Requests to access the datasets should be directed to the corresponding author at pmeagher@zoo.nsw.gov.au.
